# A protocol for an observational cohort study of heat strain and its effect on fetal wellbeing in pregnant farmers in The Gambia

**DOI:** 10.12688/wellcomeopenres.15731.2

**Published:** 2020-03-31

**Authors:** Ana Bonell, Jane Hirst, Ana M. Vicedo-Cabrera, Andy Haines, Andrew M. Prentice, Neil S. Maxwell

**Affiliations:** 1 Medical Research Council Gambia @ London School of Hygiene and Tropical Medicine, Fajara, The Gambia; 2 Nuffield Department of Women's and Reproductive Health and the George Institute for Global Health, University of Oxford, Oxford, UK; 3 Institute of Social and Preventive Medicine, University of Bern, Bern, Switzerland; 4 Oeschger Center for Climate Change Research, University of Bern, Bern, Switzerland; 5 Department of Public Health, Environment and Society; Department of Population health, London School of Hygiene and Tropical Medicine, London, UK; 6 Environmental Extremes Laboratory, University of Brighton, Brighton, UK

**Keywords:** heat stress, pregnancy, climate change, maternal, subsistence farmer

## Abstract

**Introduction: **Climate change predictions indicate that global temperatures are likely to exceed those seen in the last 200,000 years, rising by around 4°C above pre-industrial levels by 2100 (without effective mitigation of current emission rates). In regions of the world set to experience extreme temperatures, women often work outside in agriculture even during pregnancy. The implications of heat strain in pregnancy on maternal health and pregnancy outcome are not well understood. This protocol describes a study to assess the physiological response of pregnant women to environmental heat stress and the immediate effect this has on fetal wellbeing.

**Methods and analysis: **The study will be performed in West Kiang district, The Gambia; a semi-arid zone in West Africa with daily maximum temperatures ranging from approximately 32 to 40°C. We will recruit 125 pregnant women of all ages who perform agricultural work during their pregnancy. Participants will be followed every two months until delivery. At each study visit fetal growth will be measured by ultrasound scan. During the course of their working day we will take the following measurements: continuous maternal physiological measurements (heart rate, respiratory rate, chest skin temperature and tri-axis accelerometer data); intermittent maternal tympanic core temperature, four point skin temperature, blood pressure; intermittent fetal heart rate and, if eligible, umbilical artery doppler; intermittent environmental measurements of air temperature, humidity, solar radiation and wind speed. Venous blood and urine will be collected at beginning and end of day for biomarkers of heat strain or fetal distress and hydration status.

## Introduction

The world is getting hotter and current projections show no sign of this warming slowing down ^1, 2^. This global increase in heat comes with increases in both number and duration of heat waves ^3^. A recent study described the “temperature of equivalence” concept, which quantified the heterogeneity of surface temperature by geographical regions and demonstrated that low-income countries will bear a greater burden of severe heat events compared to high-income countries, even if target temperatures of less than 1.5°C are met ^4, 5^.

Pregnancy is a vulnerable time. Hyperthermia in the first trimester is teratogenic ^6– 8^ and there is epidemiological evidence of increased preterm births, low birth weight (LBW) and stillbirths following maternal exposure to heat stress ^9– 13^, though data from Africa are sparse and contradictory ^14– 16^.

In temperate regions, a case-crossover study from California found an 8.6% increase in prematurity with every 5.6°C increase in ambient temperature exposure ^17^. An intra-population analysis quantified the effect of heat on birth weight and found that heat explained 9.6% of the difference in birth weight between populations ^18, 19^.

Heat stress (a combination of ambient temperature, humidity, solar radiation and wind speed) and the consequent heat strain (the physiological response to heat stress) have not been studied in pregnant women in the field. Heat strain presents as a spectrum from perceived discomfort to death ^20^. There is almost no field-based physiology studies concerning the impact of heat stress on maternal physiology or the impact that it has on the developing fetus.

### Temperature regulation

**Figure 1.**
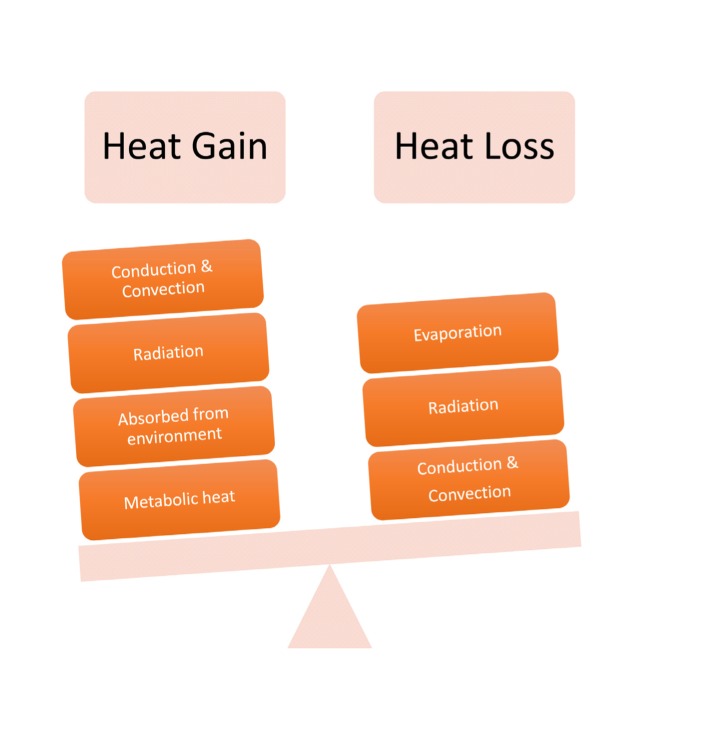
Thermal factors involved in the maintenance of the heat balance of a body.

Healthy human bodies maintain a core temperature of around 37°C ^21^. On a cellular level, this ensures an ideal environment for processes necessary for life, for example enzymes to work optimally and proteins to fold in the required configuration ^22^. Heat balance is maintained when heat is lost at a similar rate to which it is produced or absorbed, as visualized in Figure 1 ^23^.

**Figure 2.**
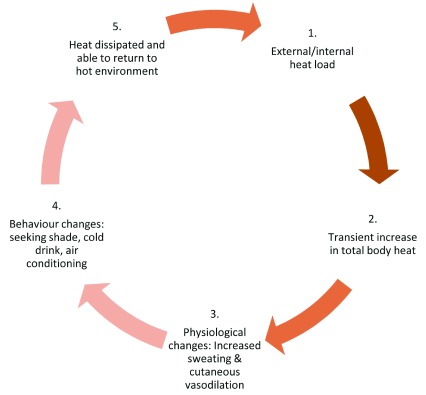
Normal physiological response to heat.

Heat loss can be altered by two mechanisms, autonomic thermoregulation where physiological processes take place without conscious effort, or behavioural thermoregulation, where conscious action is used to reduce body temperature. In certain situations the heat burden cannot be entirely avoided, for example agricultural workers and therefore in these situations the physiological mechanisms act to try and ensure heat balance is maintained. Thermal homeostasis is controlled by the preoptic anterior hypothalamus which receives afferent signals from thermal sensors in the skin and visceral core ^24^. The efferent signals stimulate cutaneous vasodilation and sweating. These increase conductive, convective, radiative and evaporative heat loss, see Figure 2.

In contrast to behavioural thermoregulation, which has a near-infinite capacity to regulate body temperature, physiological responses to environmental heat stress have a finite capacity ^25^. Heat acclimation (physiological adaptations due to repeated laboratory based heat training) and acclimatization (physiological adaptations due to repeated exposure to heat in the natural environment) improves a body’s response to heat stress; however, at a certain point even these mechanisms will be overwhelmed and core body temperature will rise ^26, 27^.

When the internal heat production increases due to increased metabolic demand and/or mechanical work, there is a delay in the body’s response to the additional heat stress. On average it takes 45 minutes for a body to reach equilibrium, and prior to this there is heat storage in the body ^27^. This is also followed by a post-exercise attenuation of heat dissipation, such that it may take 2 hours for a body to return to thermal equilibrium after exercise ^28^. The impact of this on maternal and fetal physiology and health is unknown.

### Pathophysiology of heat strain

**Figure 3.**
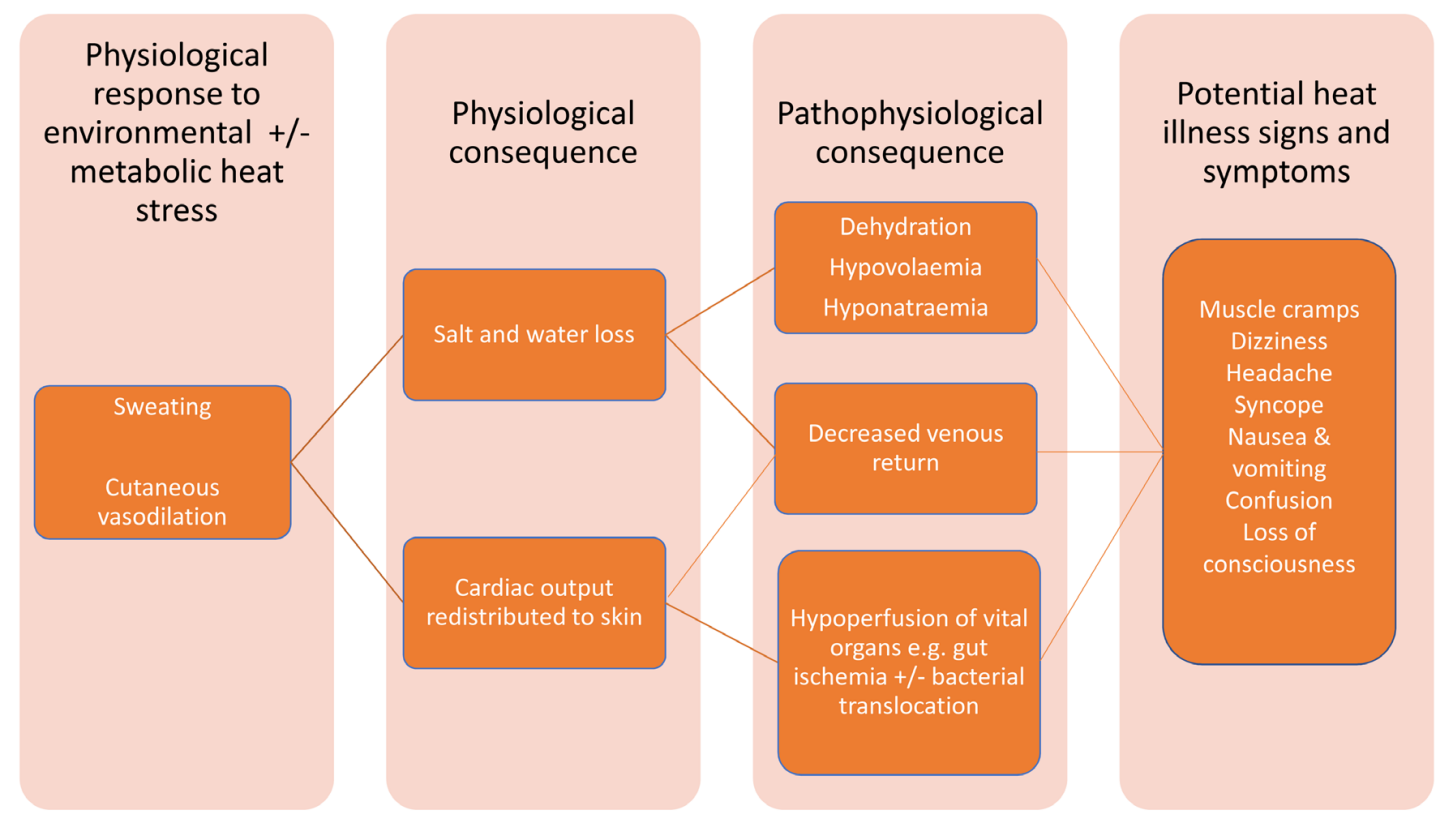
Pathophysiology of heat strain.

When a body’s capabilities to alleviate heat stress by thermoregulatory mechanisms are overwhelmed then heat strain develops, see Figure 3.

When cutaneous vasodilation is stimulated, blood supply to the skin increases from around 1% of cardiac output to as high as 70% (6-8 L/min) ^24, 29^. This necessitates a large reduction in blood flow to internal organs, in particular the splanchnic and renal arteries, as well as a reduced venous return. If the heat stress continues, then hypovolaemia due to water and salt loss in sweat exacerbates the reduction in cardiac output and the consequent reduction in blood supply to internal organs. If this continues, there is a risk of acute kidney injury, and splanchnic and cerebral blood flow insufficiency. Interruption of the splanchnic blood flow has been shown to result in ischemia of the gastrointestinal membrane, which potentially results in translocation of gut bacteria and endotoxins ^30^. If the heat stimulus is removed at this point, there is still the risk of developing systemic inflammatory response syndrome (SIRS), acute respiratory distress syndrome (ARDS), disseminated intravascular coagulopathy (DIC), multi-organ failure and death due to the stimulation of the pro-inflammatory cascade ^31^.

### Specific considerations in pregnancy

During pregnancy, maternal physiological changes are dramatic. Plasma volume increases by almost 50% in the third trimester, red blood cells increase by a lesser extent, giving a dilutional anaemia, and cardiac output also increases by around 50% ^32, 33^. The placental blood flow in the third trimester is 600–700 mL/min and is regulated by local vasoactive mechanisms rather than central neuronal command ^34^.

In terms of thermal regulation, thermal capacity increases, core temperature decreases, heat production increases and surface-area-to-volume ratio decreases over the course of pregnancy ^35^. Although some of these act to protect the pregnant woman, the overall impact of these mechanisms is a reduction in the ability to dissipate heat and an increased risk of heat strain in pregnancy. The fetal heat balance is dependent on fetal heat generation (metabolic rate), maternal temperature and uterine blood flow; however, the fetus itself has no ability to actively loose heat ^8^. Heat loss occurs mainly through the umbilical artery, although some heat is lost to the amniotic fluid. The fetus is usually 0.4-0.6°C hotter than maternal core temperature, but in situations where maternal core temperature rises, this will result in heat transfer to the fetus ^36, 37^. The impact of heat on fetal development has been a difficult area to study. Women with pyrexia, usually from an infection, experience several other factors that affect the fetus, namely microbial factors, immune responses and maternal physiological response. Owing to the difficulty in isolating the effect of heat strain in human pregnancy little is known about the changes in placental blood flow, release of heat shock proteins and other chemical responses to heat strain and what these mean for fetal wellbeing. What is known is largely taken from the animal literature and heat stress has been shown to reduce birth weight in a variety of mammals. In particular, a large body of work has examined heat stress in ewes, where placental weight and size was diminished, blood flow to the uterine artery reduced and intrauterine growth retardation (IUGR), similar to stunting, was seen in animals in a chronic heat stress environment ^38– 41^. The impact of heat stress varied with trimester, with increased rates of first trimester miscarriage and congenital abnormalities. Heat stress in the second and third trimesters resulted in IUGR and increased the incidence of stillbirth ^42^. These studies give an insight into what may be occurring in humans; however, in many cases, it is difficult to directly transfer to the human condition due to large differences in the volume to surface area ratio, and in the relative mass of the products of conception. Consequently, the pregnant ewe has often be considered to be the optimal animal model but still has important differences (the rumen and fleece for instance) and therefore conclusions must be viewed with caution.

**Figure 4.**
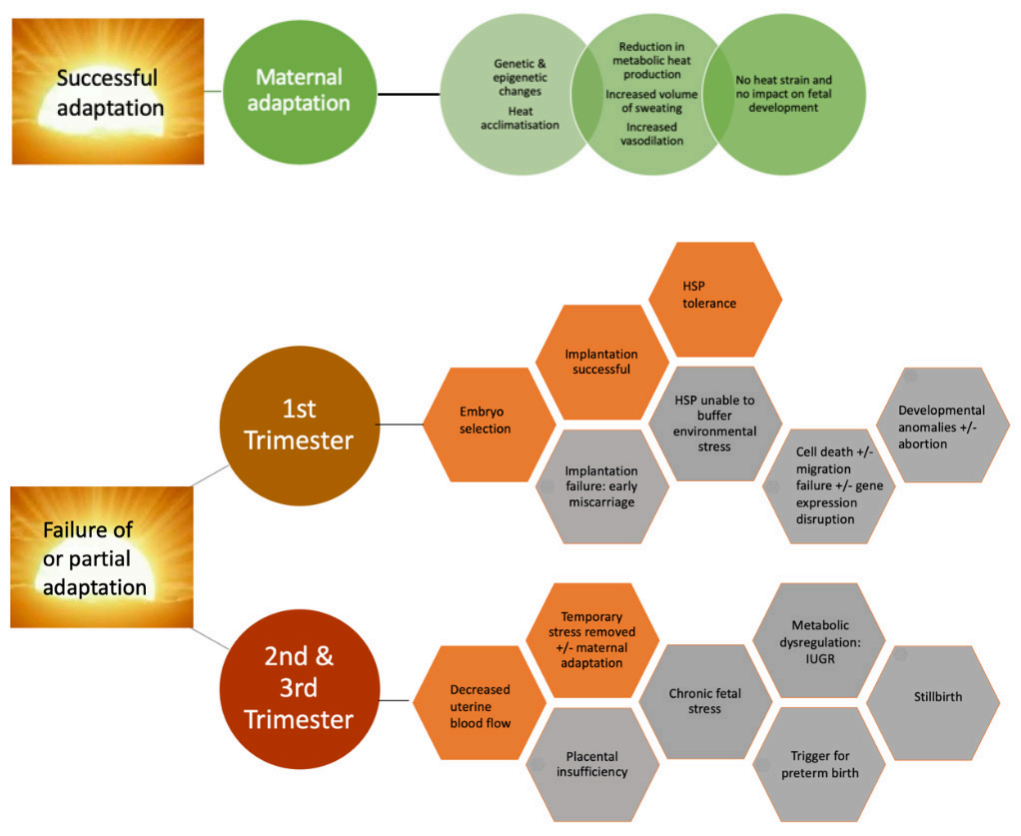
Theoretical framework of impact of heat stress on pregnancy. Orange hexagons indicate physiological impact of heat that does not necessary result in harm. Grey hexagons indicate harmful changes to the fetus. HSP, heat shock protein, IUGR, intrauterine growth retardation.

Figure 4 gives an overview of the hypothetical impact of heat stress by trimester.

### Climate change, occupational health and pregnancy

The knowledge gap relating to pregnancy in humans and exposure to heat stress is of current and growing concern as present conditions can be extreme for pregnant women and climate change predictions put the global temperature at levels not experienced in the last 200,000 years (i.e. the timespan modern humans have inhabited the earth). The burden of that heat stress will be mostly felt in low income countries with the least opportunity for adaptation ^5^. It will also occur in areas where women make up almost 50% of the agricultural work force and work throughout pregnancy ^43, 44^.

### Aims and objectives

We aim to assess whether heat strain in pregnant farmers in The Gambia acutely alter fetal wellbeing. The aims of this study are to:

1. Determine the heat stress exposure of pregnant farmers.
2. Determine the prevalence of heat strain by trimester and heat stress exposure in pregnant farmers.
3. Determine if biomarkers of heat strain correlate with physiological measurements in pregnant farmers.
4. Determine if maternal heat strain has an immediate impact on fetal heart rate or blood flow as an indication of fetal wellbeing.
5. Determine if biomarkers of feto-placental function are altered by maternal heat strain.

## Methods

### Study design

This is a prospective observational cohort study of pregnant women who perform outdoor agricultural work during pregnancy, which has been recruiting since August 2019.

### Setting

This study will be conducted at Keneba field station, Medical Research Council Unit The Gambia at London School of Hygiene and Tropical Medicine (MRCG @ LSHTM).

MRC Keneba is a rural field station based 2.5 hours inland from the coast, in Kiang West region where mostly subsistence agriculture is practiced. The climate in this area has two distinct seasons, the wet and dry season, which run from July to October and November to July, respectively. Farming of rice and groundnuts occur during the wet season and relies mostly on rainfall. In the dry season there are large “gardens”, which are used to grow a variety of vegetables. These tend to be watered by hand. Farming is a gender specific activity, with men growing the cash crops and women mainly growing food for household consumption or selling at the local markets. All agricultural work practiced by women in the region is done manually – planting, transplanting, weeding, harvesting, clearing, tilling and watering. Previous work in The Gambia has assessed the energy expenditure of pregnant women during different agricultural activities and also assessed the amount of time spent on these activities. These studies show that women will work between 50% to 83% of a 9-hour day on agricultural work, depending on the season, even when pregnant ^45– 48^.

The mean monthly temperatures in 2017 varied from 25.4–30.3°C and maximum monthly temperatures from 31.5–39.5°C. The maximum monthly Wet Bulb Globe Temperature (WBGT) varied from 24.7–29.3°C. This gives the exposure during the hottest times of the day. This exposure is at a level that international guidelines would identify as at risk of heat illness. The annual average temperature rise since 1980 is just below 1°C. Most villages do not have electricity and therefore no access to air conditioning or electric fans. Water is mostly supplied through public bore holes, although some homes do have tapped water.

### Participants and recruitment

Community sensitization and discussion will occur in each village prior to any visits. Once agreement and consent from the village elders has been obtained, we will recruit 125 participants.

Pregnant women will be approached and informed about the study in their preferred language. Inclusion and exclusion criteria are set out below.

Inclusion criteria:

1. Confirmed pregnancy with live single fetus
2. Provision of written informed of consent or witnessed thumbprint
3. Live and work within the region
4. Spend time during pregnancy in any of the following activities; working as an agricultural labourer; outside labour on a small-hold farm; gardening for at least 3 hours
5. Willingness and ability to provide demographic and clinical information, blood and urine samples and wear a non-invasive portable device for continuous physiological monitoring

Exclusion criteria:

1. In immediate need of medical attention or emergency obstetric care
2. Diagnosed with pre-eclampsia or gestational diabetes in this pregnancy
3. History of cardiac disease

### Sample size

Previous studies on physiological changes in pregnant women working in heat have not been completed. Based on published literature, we expect around 35% of agricultural workers to experience heat strain and assume this incidence risk remains at least as high in pregnancy ^49, 50^. Assuming an unexposed incidence risk of fetal distress to be 5% with an alpha of 0.05, we will need to recruit 99 participants to be powered to detect an exposed incidence risk of 30% with fetal distress. Taking into account drop-out rates due to fetal loss, we will recruit 125 participants.

### Study procedures

**Figure 5.**
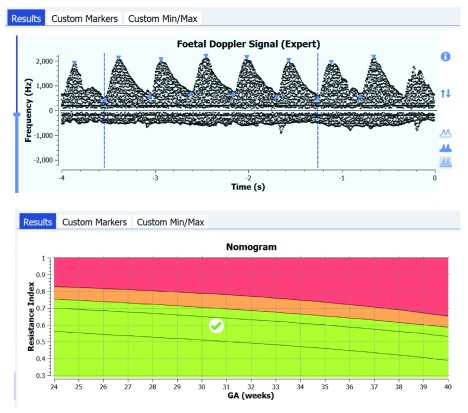
Fetal Doppler signal and associated normogram produced when using the UmbiFlow™ device.

Pregnant women, of any gestation, identified by the demographic surveillance system (DSS), antenatal clinics or village assistants will be approached and consented if eligible. They will attend the Keneba antenatal clinic where socio-economic, demographic, medical and obstetric details will be collected. These will include any past medical history, past obstetric history including previous miscarriages, stillbirths, premature births or low birth weight infants. A baseline ultrasound will be performed by a trained member of staff. Gestational age will be determined based on an early ultrasound scan (under 28 weeks gestation) using biparietal diameter. If an early ultrasound scan has not been performed then biparietal diameter will still be used but with the expectation of reduced accuracy in the estimation of gestation. In women between 28–34 weeks we will perform an UmbiFlow™ scan. The UmbiFlow™ device was designed in South Africa for use in low-resource settings to identify women at risk of poor birth outcomes due to placental pathology. It measures the resistance index (RI) in the umbilical artery and plots this on a normogram based on gestational age (see Figure 5 for an example). This device is designed for use by unskilled practitioners and requires minimal training. It has been validated for gestational ages 28–34 weeks ^51, 52^.

Within the next 2 weeks, on the day they are working outside, they will attend Keneba field station where they will have baseline anthropometry, physiology readings and blood and urine collection. They will be fitted with an Equivital™ LifeMonitor device. This is a portable, multi-parameter telemetry device that sits within a Lycra chest belt with inbuilt fabric sensors ^53^. Once wearing the LifeMonitor device they will complete a 6-minute walk test to determine cardiovascular reserve and calibrate the device ^54^.

**Figure 6.**
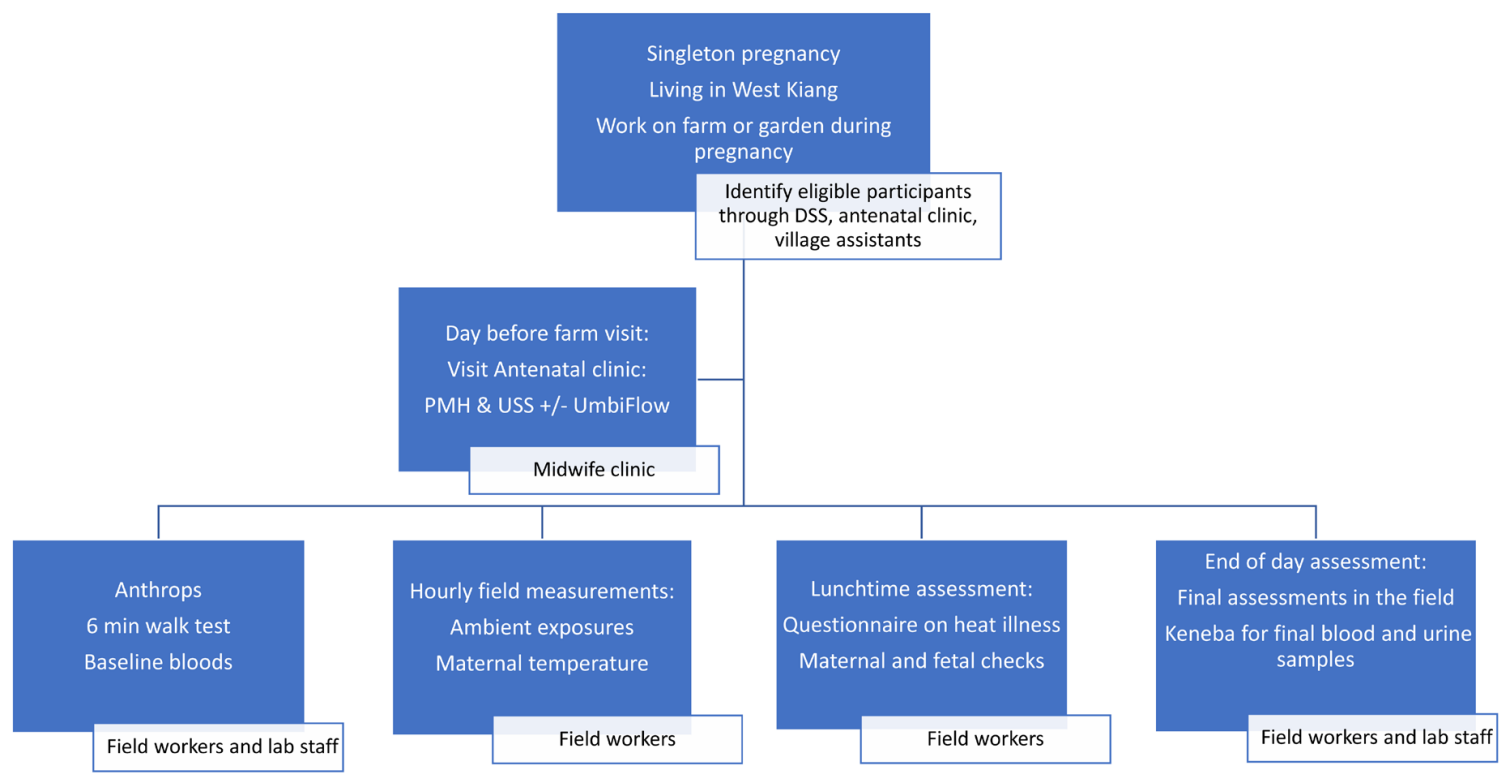
Study scheduling. DSS, demographic surveillance survey; PMH, past medical history; USS, ultrasound scan.

**Figure 7.**
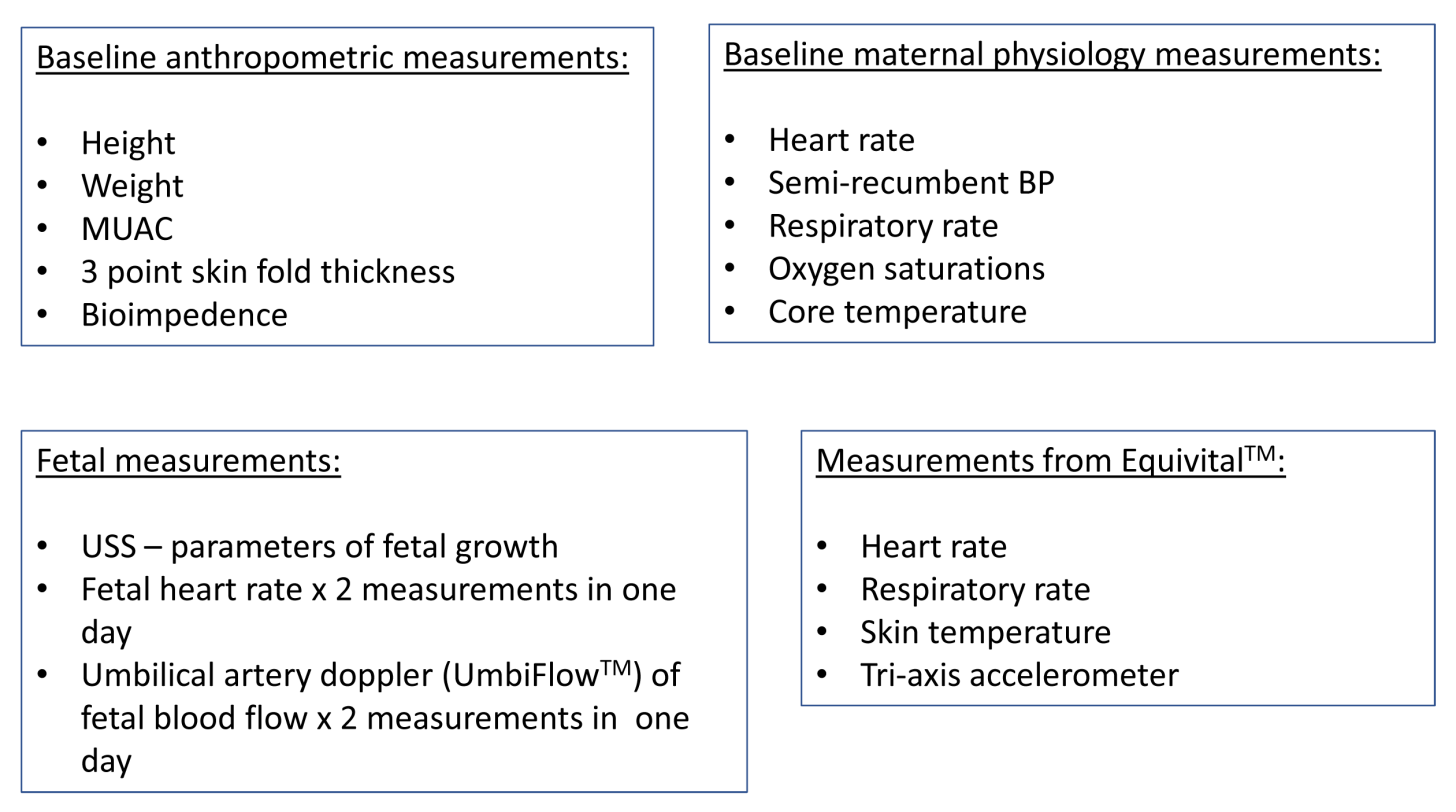
Data collection of anthropocentric, maternal, fetal and physiological measures. MUAC, mid-upper arm circumference; BP, blood pressure; USS, ultrasound scan.

During the working day (duration recorded) we will record their tympanic temperature and the ambient conditions every hour. At middle and end of day we will assess fetal heart rate ± umbilical artery flow. Maternal measurements will include a four-point skin temperature using an infrared, non-contact thermometer. Measurements are taken from four-left-hand sided points from 20 cm away; chest, mid-tricep, mid-thigh and mid-calf ^55^. A Perfect ^®^Prime thermal imaging camera IR10019 with a resolution of 320 x 240 and pixels of 76,800 will be used to take two pictures per time point; from the waist up (with head-dress removed), and from the waist down (with legs revealed). Heart rate and blood pressure will be measured with an automatic OMRON M3 Intellisense device. Standardised ratings for thermal sensation and comfort will be recorded. At the end of the participant’s normal working day we will collect end line blood and urine and take a final measurement of weight and bioimpedence. Participants will be followed every 2 months during the course of their pregnancy. Figure 6 and Figure 7 give an overview of study processes and timing.

After delivery, data will be collected on birth outcome, birth weight, gestational age, infant sex and maternal and newborn status.

Recruitment will be over a 12-month period to ensure different seasonal exposure to work and heat. By recruiting over the course of a year and repeating measures every two months we will capture different trimesters for the same women. This will give us an estimate of the physiological changes that occur at different heat exposures and by different trimesters and identify if these alterations lead to altered fetal wellbeing.

### Primary outcome measures

The primary outcome is a measurement of fetal distress. We define compromised fetal wellbeing as either: (i) a baseline fetal heart rate above 160 bpm or below 115 bpm; and/or (ii) if the fetus is 28–34 weeks gestation, then UmbiFlow™ above the 75th percentile of established resistance index graphs, or absent end diastolic flow, in keeping with the findings from South Africa and the developers of UmbiFlow™ ^51, 52^.

### Laboratory sample collection and processing

Study staff will collect a venous blood and urine sample for each participant for use in study laboratory procedures. All samples aim to identify maternal heat strain or fetal wellbeing. Table 1 gives the laboratory sampling and justification. Whole blood samples from each participant will be used to prepare six dried blood spots of 10 µl each on filter paper and stored for biomarker testing. Serum samples will be separated and stored at -80°C for future analysis.

**Table 1.** Laboratory tests.

Investigation	Purpose of the test	When to be taken
Haemoglobin & haematocrit	Identify if anaemic Indication of change in hydration status during activity	Beginning and end of day
Urea & creatinine	Indication of renal function and hydration status	Beginning of day
CRP	Inflammatory markers that are known to alter acutely in heat strain and/or in fetal distress ^59– 62^	Beginning and end of day
IL-6, IL-8, IL-10		
TNF		
Lipopolysaccharide	Increases when gastrointestinal permeability increases in heat strain ^29, 30, 63^	
Intestinal fatty acid binding protein		
Heat shock protein 70	Intra and extracellular heat shock proteins are altered in heat strain and may play a role in placental function ^56, 64, 65^	
Glucose	Alter in response to physiological stress of exercise and/or heat	
Cortisol		
Urine specific gravity and osmolality	Indication of hydration status	Beginning and end of day
Alphafetoprotein, apoliprotein C-II & III	Indication of placental function ^59^	Beginning and end of day

CRP- C-reactive protein; IL-6 interleukin-6; TNF, tumor necrosis factor.

### Statistical analyses

Statistical analysis will be performed using R. Appropriate descriptive analysis will be used to present maternal characteristics and environmental heat stress exposures. Data will be assessed for normality and skewed data will be appropriately transformed.

### Derived values

Metabolic rate and energy expenditure will be determined from the raw accelerometer and heart rate data using complex non-linear modelling. The 6-minute calibration test will allow development of individual and trimester specific estimates of metabolic rate. These will be cross-checked against historic data on energy expenditure of pregnant women in West Kiang per activity type.

Heat strain will be determined by either the physiological strain index (PSI) or the Center for Disease Control (CDC) recommended signs and symptoms score.

The PSI model is based on changes in heart rate and core body temperature and therefore gives an indication of the combined thermal and cardiovascular load:

$$\text{PSI}=5\times ({\text{T}}_{\text{core}1}-{\text{T}}_{\text{core}0})/(39.5-{\text{T}}_{\text{core}0}))+(5\times ({\text{HR}}_{1}-{\text{HR}}_{0})\times (180-{\text{HR}}_{0}))$$

Where 0 indicates baseline and 1 indicates rate or value during exposure ^20^. This has been used in multiple studies on physiological changes in exercise and/or heat but not in pregnancy ^56, 57^.

The CDC method is based on a series of symptoms related to heat illness, which vary from heat rash to heat stroke ^58^. We will include those related to heat stroke, heat exhaustion and heat cramps or a core temperature above 38°C, but will not include symptoms of heat rash or sunburn as these are not related to the physiological changes we are interested in. There are several heat stress indices we will calculate based on the direct field measurement we will take. These will include the WBGT, the Universal Thermal Climate Index, the apparent temperature and the heat index.

### Primary outcome analysis

A mixed-effect linear model will be run, using lme4 package in R to allow fixed and random effects to be incorporated appropriately.

The expected final model will be of the form: Fetal distress(ij) = fixed part [heat stress index + PSI/heat strain + maternal age + gestational month (or trimester) + nutritional status + metabolic rate + cardiac reserve + heat illness symptoms + ΔHct + Δbioimpedence] + random term [individual participant]

Fetal compromise(ij) = presence or absence of fetal distress as defined above for individual i at gestational month j (1…9).

### Secondary outcome analysis

Different commonly used heat stress indices as described above will be validated against heat strain data for clinical correlation. Changes in fetal heart rate from baseline, stratified by trimester will be explored. Heart rates > 170 and > 180 will also be used as cut offs for fetal distress, although the numbers may be small. Changes in biomarkers of heat strain or feto-placental function will be analysed by ANOVA stratified by trimester and heat stress exposure.

### Safety and ethical considerations

This study has been approved by the Gambia government/MRC Joint ethics committee (ref: 16405) and the London School of Hygiene and Tropical Medicine Ethics Advisory Board.

Written informed consent and information sheets will be provided to all participants. A trained study staff member will conduct individual screening interviews and informed consent procedures in the preferred language of the participant. If the participant is unable to write, her fingerprint will be used as substitute for a signature, and an impartial adult witness to the entire consent procedure will provide their signature.

Potential participants will be able to ask questions and discuss the study with study staff at any time during and after study activities. Participants are free to withdraw consent at any time during the course of the study and this will not impact on future care provision.

Risks associated with participating in this study are minimal. Participants will be screened at the start of the day and should they demonstrate any signs or symptoms of illness or concern they will be advised to seek the attention of the Keneba health clinic and participation in the study will be delayed until they are well. Should a participant be hypertensive but not pre-eclamptic then may still enter the study, but we will refer them to antenatal services for treatment of their hypertension. Should a participant develop pre-eclampsia after recruitment, diagnosed at antenatal clinic or on subsequent visits, they will be referred to Keneba antenatal services and not included in the daily assessment of maternal heat strain and fetal wellbeing. However, if they are willing to remain in the study, pregnancy outcome data will still be collected.

Participants will have additional venous blood samples taken using aseptic technique with universal precautions to minimize the risk of infection, personal discomfort, transient bleeding and bruising that may result. An ultrasound scan is part of routine antenatal care and an additional scan adds no harm to maternal or fetal health. The risks of wearing the portable recording devices include chaffing of the skin and discomfort, which we will minimize by ensuring a good fit at the beginning of the day and checking for any skin irritation at the end of the day.

During the 6-minute walk test the participant can stop at any point during the course of the exercise and standardised feedback was collected. Additionally, this test will be performed at Keneba field station, close to the clinic area and if any untoward symptoms are experienced, they will assess and treat the participant as required.

During the course of the day, if a significant heat load is experienced and the maternal core temperature increases beyond 38.5 ^o^C (see below) the guidelines on treatment of heat strain will be followed with some additional considerations. In non-pregnant individuals, heat strain is determined to be a life-threatening emergency requiring immediate treatment when core temperature reaches 40.5°C ^31^. In pregnancy, heat is known to be teratogenic in the embryonic period, and throughout pregnancy compensatory mechanisms may be compromised. Hence we do not think it is ethical to allow the temperature to reach such a high level. Therefore, should maternal core temperature reach 38.5°C, this would result in an immediate review and treatment of heat strain would be commenced. This would include an overall clinical assessment of the women and fetus, immediate measures to treat the women and if these did not result in improvement within 30 minutes, consideration of transfer to the health facility.

If during the intermittent measurements of fetal wellbeing, there are any concerns with fetal heart rate (either >160 or <115) ^66^, or regarding the Doppler results, then the participant will be assessed, encouraged to rest in the left lateral position, consume water, and have observations of fetal movement and maternal blood pressure taken. If after 30 minutes, the baseline heart rate has not returned to the normal range, the Doppler remains abnormal, or there are any clinical concerns, the women will be offered transport to the health clinic for further review and treatment. Any such events will be recorded as an incident case of fetal distress as per the primary outcome of this study.

### Limitations

This is a field-based study in rural West Africa and therefore there are several limitations when comparing it to a laboratory based heat chamber study. We aim to characterize the physiological response of pregnant women to heat stress. Due to ethical considerations, this is an observational study only. Therefore if the women are not exposed to extreme heat in their usual work, we will not be able to measure this effect. However, from previous work in this setting, women were working in the heat during pregnancy and therefore we are confident that the exposure will occur.

Since we are recruiting women who work in the gardens or farms during pregnancy, we are aware that our sample may be biased towards those in the lowest socio-economic group, which may affect the generalisability of the result. However, we consider this group to be of particular importance as they are likely to have little options for modification of behaviour or development of adaptation strategies. We will use Demographic Health Surveillance Data to compare our sample with the wider pregnant community over the time period of our study to determine the representation in the sample and any significant sources of bias.

Our physiology measurements do not include continuous core temperature monitoring as would be the gold standard, due to practical constraints of field work. We therefore use tympanic temperature as a measure of core temperature, recognising the deficiencies in this measurement. Due to well documented measurement errors in this method, we are likely to underestimate the true temperature rise. Additionally the Equivital device measurements, heart rate, respiratory rate, skin temperature and tri-axis accelerometer will all have measurement errors. We will attempt to minimise these by removing impossible values, cleaning the signal and using the inbuilt algorithms for percentage confidence in the results.

Due to the practicalities of accessing remote rice fields, and transporting all necessary equipment we will be unable to accurately assess water intake. Our assessment of hydration therefore is at the physiological level, but this is a limitation in the methodology especially considering that hydration is an important aspect of thermoregulation.

### Confidentiality and access to data

All participants will be allocated a unique identifying number (UIN) at recruitment. Data generated by the wearable sensors will be downloaded from the devices at the end of the day, linked to the UIN and then wiped. During the study day, data will be collected on tablets using the REDCap application. On return to Keneba field station the tablets are synced, allowing transfer of encrypted data to the designated server.

All data will be backed up regularly by the IT department in accordance with MRC SOP-INT-001. The database is centrally stored, data is secure and encrypted and held by MRC/LSHTM. No personal identifiable information will be available in any shared or published document. Primary data outputs will be in XML format. All study documents will be filed and stored for at least 10 years.

### Dissemination

The results of the study will be analysed and prepared for publication in open-access peer-reviewed international journals, staggered over time. At the end of the project a community event will be held to disseminate results to all those communities that participated in the study. We will comply with international standards and guidelines regarding open access of research data.

## Conclusion

This study will be the first to characterize the heat stress, heat strain and fetal status in pregnant farmers and with these results we hope to describe the problem, measure the incidence of significant threats to fetal well-being, and to highlight the need for ongoing work in this area with an ultimate aim of developing adaptation interventions to mitigate the problem.

## Data availability

No data are associated with this article.
