# Supplementary material for: A protocol for an observational cohort study of heat strain and its effect on fetal wellbeing in pregnant farmers in The Gambia
Source: Wellcome Open Res. 2020 Mar 31;5:32. Originally published 2020 Feb 18. [Version 2] doi: 10.12688/wellcomeopenres.15731.2 (PMC7141168; doi:10.12688/wellcomeopenres.15731.2)
Supplement: Peer review history

# Peer review: Reviewer response for version 2

**DOI:** 10.21956/wellcomeopenres.17328.r38292

**Published:** 2020-04-07

**Authors:** Beth Holder

**Affiliations:** 1 Institute of Reproductive and Developmental Biology, Department of Metabolism, Digestion and Reproduction, Imperial College London, London, UK

I am happy with the response and revisions, and approve this study for indexing.

I confirm that I have read this submission and believe that I have an appropriate level of expertise to confirm that it is of an acceptable scientific standard.

# Peer review: Reviewer response for version 1

**DOI:** 10.21956/wellcomeopenres.17244.r37960

**Published:** 2020-03-09

**Authors:** Beth Holder

**Affiliations:** 1 Institute of Reproductive and Developmental Biology, Department of Metabolism, Digestion and Reproduction, Imperial College London, London, UK

This is an ambitious and timely study, particularly given growing interest in the effects of extreme heat on pregnancy outcome. The Keneba field station in The Gambia is an ideal place to run this study, and the planned wide-range measurements will address some important questions and generate useful data. The overall study design is very good, with the following caveats/considerations: 

1. More information is needed on the exact gestation that will be studied. This should be clearly stated in the inclusion/exclusion criteria. It is not stated when women will be approached and consented, and hence what gestation the 2-week study period will cover. It is only mentioned that the UmbiFlow will be performed on women between 28-34 weeks. Is this the target gestational period that will be studied?
2. Related to the above, including a wide range of gestations could impact on the required sample sizes, as gestation could play an impact on either physical exertion (i.e. the mother resting more in later gestation), or on the sensitivity of the fetus to stress. I would like to know a bit more about the range of gestation that will be studied, and how gestational age will be considered in the analysis (i.e. will stratification be performed).
3. Will liquid intake be monitored? This could have an impact on maternal wellbeing, and also impact on urine and blood analyses. Perhaps water could be provided in reusable bottles, and the amount left over at the end of the day recorded.
4. Will blood samples at the beginning of the day be fasted samples, or after eating? Same for the end of the day sample - will the time since their last meal be recorded?
5. Normal fetal heart rate varies with gestation. It would be good to consider this in the study. It would also be good to include HR >170 and >180 in the secondary analysis.
6. Accurate estimation of gestation could be quite important in this study. It is not clear how often early ultrasounds will be performed. Has capability been built in this area? Are there designated individuals who will perform ultrasounds?
7. Related to the above, I am concerned that LMP and biparietal diameter (BPD) will be used interchangeably. In how many women is LMP expected to be used? Will LMP also be recorded for those with BPD measurements to see how they compare?
8. How will the gestation at ultrasound scan be considered? My understanding is that at 12-26 weeks, it is accurate +/- 10-11 days, and after 27 weeks, it is accurate +/- 2-3 weeks. It would be good to give some detail on the limitations of using a single BPD measurement. If possible, paired BPD measurements at two timepoints would improve the accuracy of estimating gestational age.
9. Will hypertensive women be included? It is not mentioned in the exclusion criteria, but presumably anyone who is identified as hypertensive at time of consent will be excluded?
10. Will participants be blinded to their measurements, or will it be fed back to them each day? I wonder how they many adapt their behaviour in subsequent days if they are aware of their previous measurements (of course exempting when there are concerns of maternal/fetal wellbeing, which will rightly result in intervention).
11. Finally, there is not much detail provided on what clinical parameters will be recorded that may be important to consider in any analysis. E.g. maternal age, ethnicity, BMI, parity, gravidity, previous pregnancy pathologies (e.g. miscarriage, stillbirth, pre-eclampsia, preterm birth, gestational diabetes).

I confirm that I have read this submission and believe that I have an appropriate level of expertise to confirm that it is of an acceptable scientific standard, however I have significant reservations, as outlined above.

# Response

**Published:** 2020-03-18

**Authors:** Ana Bonell

**Affiliations:** London School of Hygiene & Tropical Medicine, UK

Thank you for your time and effort in providing the feedback on the paper. We are very grateful for the scientific review and hope that the changes we have made in response to the suggestions make the article stronger scientifically.

 1. More information is needed on the exact gestation that will be studied. This should be clearly stated in the inclusion/exclusion criteria. It is not stated when women will be approached and consented, and hence what gestation the 2-week study period will cover. It is only mentioned that the UmbiFlow will be performed on women between 28-34 weeks. Is this the target gestational period that will be studied?

 We decided to include any gestation. Since we repeat our field measurements every two months, we hope to get at least 2 visits per participant. Due to the variation in climate exposures we did not want to limit the gestation we would explore at the start of the project since those environmental conditions (hot and humid) only last 3 months. If we limited the recruitment to early gestation then we would not have late pregnancies exposed to those conditions. This has been made clearer in the manuscript.

 2. Related to the above, including a wide range of gestations could impact on the required sample sizes, as gestation could play an impact on either physical exertion (i.e. the mother resting more in later gestation), or on the sensitivity of the fetus to stress. I would like to know a bit more about the range of gestation that will be studied, and how gestational age will be considered in the analysis (i.e. will stratification be performed).

 Gestation will be included in the analysis. We will begin by exploring the results stratified by trimester, but will consider using a fixed effect of gestational weeks or months in the full model. Stratification by trimester will be used for analysis of heat strain, biochemistry changes and changes in osmolality.

 This has been made clearer in the manuscript

 3. Will liquid intake be monitored? This could have an impact on maternal wellbeing, and also impact on urine and blood analyses. Perhaps water could be provided in reusable bottles, and the amount left over at the end of the day recorded.

 This is a very important point. Unfortunately our study has taken us to remote areas where our 4WD is unable to reach. We have then had to carry all our equipment out into the field. Due to these constraints we have been unable to do this.

 I will add this as a limitation

 4. Will blood samples at the beginning of the day be fasted samples, or after eating? Same for the end of the day sample - will the time since their last meal be recorded?

 Blood samples will not be taken fasted

 5. Normal fetal heart rate varies with gestation. It would be good to consider this in the study. It would also be good to include HR >170 and >180 in the secondary analysis.

 This will be added to our secondary analysis. Additionally we will also look at the change in fetal heart rate from at rest in a cool room to when working in the field and so use individual resting rates as our baseline.

 6. Accurate estimation of gestation could be quite important in this study. It is not clear how often early ultrasounds will be performed. Has capability been built in this area? Are there designated individuals who will perform ultrasounds?

 Ultrasound scans will be performed by 1 midwife or 1 doctor who have undergone training in fetal scanning and dating measurements.

 This has been clarified in the manuscript

 7. Related to the above, I am concerned that LMP and biparietal diameter (BPD) will be used interchangeably. In how many women is LMP expected to be used? Will LMP also be recorded for those with BPD measurements to see how they compare?

 This has been altered as you suggest. LMP is very unreliable in this setting. Therefore we will use BPD only for dating.

 8. How will the gestation at ultrasound scan be considered? My understanding is that at 12-26 weeks, it is accurate +/- 10-11 days, and after 27 weeks, it is accurate +/- 2-3 weeks. It would be good to give some detail on the limitations of using a single BPD measurement. If possible, paired BPD measurements at two timepoints would improve the accuracy of estimating gestational age.

 Gestational dating will be based on ultrasound. Most participants will have had an ultrasound prior to 28 weeks (giving an accuracy to +/- 14 days). If the participant has not been identified early enough then this will be recorded and gestational dating will be based on BPD but with the caveat of reduced accuracy.

 This has been altered in the protocol to make clearer

 9. Will hypertensive women be included? It is not mentioned in the exclusion criteria, but presumably anyone who is identified as hypertensive at time of consent will be excluded?

 If participants are hypertensive but not pre-eclamptic they will be included. If they develop pre-eclampsia then they will be excluded.

 This has been made clearer in the manuscript

 10. Will participants be blinded to their measurements, or will it be fed back to them each day? I wonder how they many adapt their behaviour in subsequent days if they are aware of their previous measurements (of course exempting when there are concerns of maternal/fetal wellbeing, which will rightly result in intervention).

 Participants will be made aware of concerns to fetal wellbeing or raises in core temperature above 38.0 degrees. Otherwise they will not have measurements fed back to them

 11. Finally, there is not much detail provided on what clinical parameters will be recorded that may be important to consider in any analysis. E.g. maternal age, ethnicity, BMI, parity, gravidity, previous pregnancy pathologies (e.g. miscarriage, stillbirth, pre-eclampsia, preterm birth, gestational diabetes).

 All these parameters will be collected. This has been added to the manuscript to make it clear.

# Peer review: Reviewer response for version 1

**DOI:** 10.21956/wellcomeopenres.17244.r37958

**Published:** 2020-03-02

**Authors:** Mathew F. Chersich

**Affiliations:** 1 Wits Reproductive Health and HIV Institute, Faculty of Health Sciences, University of the Witwatersrand, Johannesburg, South Africa

The study covers key topic of growing concern. Pregnant women may well be now of the highest risk groups for heat exposure in LMICs, and occupational exposure is especially problematic as women may continue to work even when they feel heat stress. The biological measures are detailed, will provide much information.

 A few comments:

1. I think more is needed to distinguish between subsistence farming and farming done done for income.
2. It is also not clear if there will be a set of questions asked about heat behaviours, for example, changes in work hours as gestation progresses, changes in kinds of activities done depending on temperature, changes in behaviours, like starting earlier in the day. Changes in gender roles with gestation or temperature? Do men do more when it is hot? Is drinking water accessible?
3. There is a line which is problematic 'almost no data and no field studies concerning impact of heat stress on maternal physiology...; there is a lot written about thermoregulation in pregnancy, and it is fascinating.
4. The claim about hyperthermia being a teratogen in humans is perhaps over-stated. As I understand there is still a controversy about this in terms of saunas etc.
5. I wonder if it might be better to target the physiological measurements to days above a certain temperature threshold, based on forecasting. That is the key question I think, It depends what your question is: impact of total heat burden during pregnancy, or impact of 'extreme heat'. If it is the later then measures on days with Tmax >90th percentile might make more sense.
6. The ability of women to thermoregulate in pregnancy is remarkable I think, their relative 'hypothermia' and shifting of blood away from the fetus means that you may not detect any major impact, a major missed opportunity
7. I wonder if you need a limitations section, the one limitation under participants did not make sense to me. How much will behaviours change with the presence of a field worker? The hourly tympanic membrane measure seems excessive intrusion that may alter the behaviour you are trying to asess. I really dount the core temp will rise, certainly not by the hour! She may have heat stress and discomfort but for that to raise her temperature is unlikely I imagine.
8. The sample size refers to the 30% increases incidence in the primary outcome?  

I confirm that I have read this submission and believe that I have an appropriate level of expertise to confirm that it is of an acceptable scientific standard.

# Response

**Published:** 2020-03-18

**Authors:** Ana Bonell

**Affiliations:** London School of Hygiene & Tropical Medicine, UK

Thank you for your time and effort in providing the feedback on the paper. We are very grateful for the scientific review and hope that the changes we have made in response to the suggestions make the article stronger scientifically.

 1. I think more is needed to distinguish between subsistence farming and farming done done for income.

 In the Gambia there is a clear gender divide in farming. Men work on the cash crops and women grow what they use themselves with the potential to sell at the village markets.This has been added into the background context to aid understanding of the local situation.

  2. It is also not clear if there will be a set of questions asked about heat behaviours, for example, changes in work hours as gestation progresses, changes in kinds of activities done depending on temperature, changes in behaviours, like starting earlier in the day. Changes in gender roles with gestation or temperature? Do men do more when it is hot? Is drinking water accessible?

 These questions will not be asked as part of this study, however additional qualitative work is planned to explore these questions to complement this work.We specifically ask about heat illness symptoms, thermal comfort and sensation, thirst and perceived exertion.

 3. There is a line which is problematic 'almost no data and no field studies concerning impact of heat stress on maternal physiology...; there is a lot written about thermoregulation in pregnancy, and it is fascinating.

 I completely agree, there is a lot of data on thermoregulation in pregnancy, but field-based physiology studies in heat stressed human pregnancies are lacking. I have rephrased the sentence to more accurately reflect this.

 4. The claim about hyperthermia being a teratogen in humans is perhaps over-stated. As I understand there is still a controversy about this in terms of saunas etc.

 Hyperthermia is a teratogen in the first trimester, as shown from fever studies and studies with documented prolonged rises in core temperature.

 I have clarified that it is a teratogen in the first trimester in the manuscript.

 5. I wonder if it might be better to target the physiological measurements to days above a certain temperature threshold, based on forecasting. That is the key question I think, It depends what your question is: impact of total heat burden during pregnancy, or impact of 'extreme heat'. If it is the later then measures on days with Tmax >90th percentile might make more sense.

 This would give us a detailed idea of extreme heat. However without the ability to compare to physiological changes at cooler times it would be difficult to differentiate the heat from the exercise effects. This was the rational to recruit throughout the year.

 6. The ability of women to thermoregulate in pregnancy is remarkable I think, their relative 'hypothermia' and shifting of blood away from the fetus means that you may not detect any major impact, a major missed opportunity

 The ability to thermoregulate is impressive, however the conditions here in The Gambia are extreme in terms of what we as humans can adapt to. In particular if occupational guidelines were followed the max WBGT is often above the level at which outdoor work is advised. Therefore even with a starting relative hypothermia we expect to see some changes. Additionally, the shifting of the blood away from the fetus is a very important physiological change to document as a starting point to understand the impact of heat in pregnancy.

 I have added detail on the max WBGT we expect the women to be exposed to and how this puts them at risk of heat illness according to the ACGIH

 7. I wonder if you need a limitations section, the one limitation under participants did not make sense to me. How much will behaviours change with the presence of a field worker? The hourly tympanic membrane measure seems excessive intrusion that may alter the behaviour you are trying to asess. I really dount the core temp will rise, certainly not by the hour! She may have heat stress and discomfort but for that to raise her temperature is unlikely I imagine.

 A limitations section has been added.

 The taking of hourly tympanic membrane measurements was a compromise as we were unable to measure continuous core temperature as would have been the gold standard in heat strain studies. Rectal temperature was impossible and core telemetry pills have yet to be proved safe in pregnancy. In heat chamber studies, volunteers will put their core temperature up within 20 mins of exercise in a hot/humid environment and this will return to baseline relatively quickly which is the rational for the hourly measurements.

 8. The sample size refers to the 30% increases incidence in the primary outcome?  

 Yes, this is for the primary outcome

 I have clarified this in the manuscript
